# Construction of a nine DNA repair-related gene prognostic classifier to predict prognosis in patients with endometrial carcinoma

**DOI:** 10.1186/s12885-020-07712-5

**Published:** 2021-01-06

**Authors:** Jinhui Liu, Pinping Jiang, Xucheng Chen, Yujie Shen, Guoliang Cui, Ziyan Ma, Shaojie Zhao, Yan Zhang

**Affiliations:** 1 Department of Gynecology, The First Affiliated Hospital of Nanjing Medical University, Nanjing, China; 2 College of Pharmacy, Nanjing Medical University, Nanjing, China; 3 Department of Otorhinolaryngology, The First Affiliated Hospital of Nanjing Medical University, Nanjing, China; 4 Department of Traditional Chinese Medicine, The First Affiliated Hospital of Nanjing Medical University, Nanjing, China; 5 University of New South Wales, Sydney, Australia; 6 Department of Gynecology and Obstetrics, Wuxi Maternal and Child Health Hospital Affiliated to Nanjing Medical University, No. 48, Huaishu Road, Wuxi, 214000 Jiangsu China

**Keywords:** Endometrial cancer, Bioinformatics analysis, DNA repair, Prognostic model

## Abstract

**Background:**

Endometrial cancer (EC) is one of the most common gynecological malignancies worldwide. However, the molecular mechanisms and the prognostic prediction for EC patients remain unclear.

**Methods:**

In the current study, we performed an in-depth analysis of over 500 patients which were obtained from the Cancer Genome Atlas (TCGA) database. The bioinformatics analysis included gene set enrichment analysis (GSEA) and Cox and lasso regression analyses to ensure overall survival (OS)-related genes, moreover, to construct a prognostic model and a nomogram for EC patients.

**Results:**

GSEA identified 4 gene sets significantly associated with EC, which are DNA repair, unfolded protein response, reactive oxygen species pathway and UV response up. Twenty-five OS-related DNA repair genes were screened out, after that, a 9-mRNA signature was constructed to measure the risk scores of patients with different outcomes. In addition, a nomogram contained the 9-mRNA model and clinical parameters was also presented to assess the prognosis. Patients with low risk were more likely to have sensitivity to paclitaxel, vinblastine, rapamycin, metformin, imatinib, Akt inhibitor and lapatinib.

**Conclusions:**

The identified highly enriched gene sets may offer a novel insight into the tumorigenesis and treatment of EC. Additionally, the constructed 9-mRNA model and the nomogram have prominent clinical implications for prognosis evaluation and specific therapy guidance for EC patients.

**Supplementary Information:**

The online version contains supplementary material available at 10.1186/s12885-020-07712-5.

## Background

Endometrial cancer (EC) is a common malignancy for female which caused over 89,000 deaths in the last year all over the word [1]. This disease is generally implied a favorable prognosis at an early stage with a high survival rate (over 95%) due to fairly frequent early vaginal bleeding. However, 30% of EC patients are diagnosed at a late stage with regional or distant metastasis, resulting in less than 20% 5-year survival rate. The current method to measure patient outcomes is to combine the preoperative clinical examination with surgical exploration, as well as pathological diagnosis after surgery. Nevertheless, a fraction of EC patients exhibit a insensitivity to chemotherapy with a high risk of cancer progression or recurrence. Given the limitations of the Federation Internationale of Gynecologie and Obstetrigue (FIGO) staging system and histological classification for the evaluation of prognosis, identifying predictive biomarkers and incorporating genetic features into the evaluation systems to help clinicians provide rational therapy and predict prognosis are truly imperative [2, 3].

DNA repair has been reported to closely participate in the pathological progression of multiple cancers [4–7]. The biological role of the process in EC development has been rarely studied. In our study, we analyzed the genomic data of EC patients acquired from the Cancer Genome Atlas (TCGA) database and investigated the pathological function of the DNA repair process. Furthermore, we constructed a 9-mRNA-based prognostic signature to evaluate the risk scores of patients and presented a nomogram for clinicians to predict the outcomes of EC patients who received reasonable treatment.

## Methods

### Data collection and processing

We integrated the FPKM format of RNA-seq expression set of 552 cancerous and 35 normal tissues and the clinical data of all EC patients from TCGA cohort to obtain 520 EC patients. Then, the 520 EC patients were randomly separated into two groups, including the training group (*n* = 312) and the testing group (*n* = 208). The training cohort was applied for constructing the prognostic model, while the testing cohort and the TCGA set were used for validation.

### Gene pathway analysis

Gene set enrichment analysis (GSEA) allows the investigation of whether certain sets of genes exhibit significant differences between two groups, while the Molecular Signatures Database (MSigDB) enables the biological or functional categorization of a given gene set according to several annotated gene sets [8]. Through GSEA and the hallmark gene sets from MSigDB, we explored the potential biological pathway exchanges between EC tumors and normal endometrial tissue from the entire EC TCGA gene set. Gene sets with *p* < 0.05 and false discovery rate (FDR) < 0.01 were considered significantly enriched and were to subject to further investigation of biological processes.

### Identification of OS-related genes and the corresponding clinical characteristics

We performed univariate, lasso and multivariate cox regression analysis to find the relationship between patient overall survival (OS) and the expression of DNA repair-related genes using “survival” and “glmnet” R packages. A gene was considered an OS-related gene candidate when the *P*-value was < 0.05 in the univariate cox regression analysis via the “Survival” R package. Next, lasso-penalized and multivariate cox regression analysis were applied for further screening, and the satisfactory mRNAs were ultimately included. We also calculated hazard ratios (HRs) and regression coefficients of each gene. The type and frequency of gene alteration, and the 50 most frequently altered neighbor genes of satisfactory genes, were presented by the cBioPortal tool [9]. The correlation among OS-related DNA repair genes and Kaplan-Meier curve plots of each gene were also displayed. The Kaplan-Meier plots of OS in the two groups were divided by the best-separation value of each hub gene. The expression levels of OS-related DNA repair genes were studied with the GEPIA website, and the protein levels were measured by the Human Protein Atlas (HPA) database [10].

### Foundation of the gene-related prognostic model

The prognostic risk score model was uesd to predict clinical outcome of EC patients, which was the combination of each optimal prognostic mRNA expression level multiplied by the relative regression coefficient weight computered from the multivariate cox regression model based on the following equation:

$$ \mathrm{Risk}\ \mathrm{Score}\ \left(\mathrm{patient}\right)=\sum \limits_i\mathrm{Coefficient}\ \left({\mathrm{mRNA}}_i\right)\times \mathrm{Expression}\ \left({\mathrm{mRNA}}_i\right) $$

According to the median risk score, all the patients from training cohort were separated into high- and low-risk groups. The Kaplan-Meier analysis of both groups were performed, and the receiver operating characteristic (ROC) curve for OS prediction was used to evaluate the specificity and sensitivity of the model [11]. Multivariate regression analysis that included the patient age, tumor stage, grade and risk score was also implemented to test the independency of the prognostic model without clinical characteristics.

### Validation of the efficacy of the prognostic risk model

We compared the testing group or entire TCGA and the EC patient risk score with the cut-off value calculated from the training group. Each patient was determined as a high-risk or low-risk case. Kaplan-Meier curve analysis, Time-dependent ROC analysis, and multivariate cox regression analysis were also employed. Additionally, we also performed stratification analysis on the basis of clinicopathological features of age, tumor stage, graede and the type of histological.

### Chemotherapeutic response estimation

The chemotherapeutic response for each sample was estimated according to the Genomics of Drug Sensitivity in Cancer (GDSC) database (https://www.cancerrxgene.org/). Eight commonly used chemotherapy drugs, including cisplatin, paclitaxel, bleomycin, vinblastine, gemcitabine, rapamycin, metformin, imatinib, Akt inhibitor and lapatinib, were selected. The estimation process was performed via the R package “pRRophetic,” where the half-maximal inhibitory concentration (IC50) of the sample was evaluated by ridge regression, and the prediction accuracy was estimated by 10-fold cross-validation on the basis of the GDSC training set. All parameters were determined by the default values excluding the batch effect of “combat” and tissue type of “all solid tumors”. The duplicate gene expression was summarized as a mean value [12].

### Building and confirmation of the nomograms

We constructed the nomogram and presented a calibrated curve that incorporated the clinical factors and mRNA signature utilizing the “rms” R package. The correctness of the nomogram was examined by checking the homogeneity index of the predicted probability with actual observation frequency. Then, we visualized the results by exhibiting the predicted prognosis and the actual prognosis of the nomogram in the calibration curve. The 45° line was considered as the best prediction.

### Clinical specimens

We analyzed 10 EnCa tissues and paired them with normal tissues in present study, and all the patients were collected at the Wuxi Maternal and Child Health Hospital Affiliated to Nanjing Medical University. Our study was approved by the Clinical Research Ethics Committee of Wuxi Maternal and Child Health Hospital Affiliated to Nanjing Medical University, and was carried out according to the Declaration of Helsinki.

### Total RNA extraction and quantitative real-time PCR analysis

TRIzol reagent (Thermo Fisher Scientific, Waltham, MA, USA) was used to extract total RNA from tissue samples, and the Agilent Bioanalyzer 2100 (Agilent Technologies, Santa Clara, CA, USA) with an RNA 6000 Nano Kit to estimate the integrity of the extracted RNA. We synthesize single-stranded complementary DNA by the reaction of High-Capacity cDNA Reverse Transcription Kit (Thermo Fisher Scientific) with the extracted RNA and then conducted real-time quantitative analysis using SYBR Green PCR Kit (Thermo Fisher Scientific). The cycle threshold (Ct) of each gene was recorded. We calculated the relative expression of the target gene with the 2^−ΔΔCt^ method. All program procedures for real-time quantitative RT-PCR (qRT-PCR) were conducted based on the instructions of manufacturer. Primer sequences for GAPDH and five hub genes are presented in Table 1.

**Table 1:** Primer sequences for six hub genes and GAPDH

Gene	Primer Sequences
TP53-F	CAGCACATGACGGAGGTTGT
TP53-R	TCATCCAAATACTCCACACGC
RFC2 -F	GTGAGCAGGCTAGAGGTCTTT
RFC2 -R	TGAGTTCCAACATGGCATCTTTG
SEC61A1-F	GGACCGCTATCACCCTCTTTA
SEC61A1-R	TCCATCAATGTGCCTCTGTTAGA
TAF10-F	GCCATATCTAACGGGGTTTACG
TAF10-R	GCACGGTTCAGGTAGTAACCAG
UMPS-F	TTGGTGACGGGTCTGTACGA
UMPS-R	GAAGACGCGGTCGAGACAC
DDB2 -F	ACCTCCGAGATTGTATTACGCC
DDB2-R	TCACATCTTCTGCTAGGACCG
GAPDH-F	ACCACAGTCCATGCCATCAC
GAPDH-R	TCTAGACGGCAGGTCAGGTC

## Results

### Gene set enrichment analysis and gene screening

**Fig. 1:**
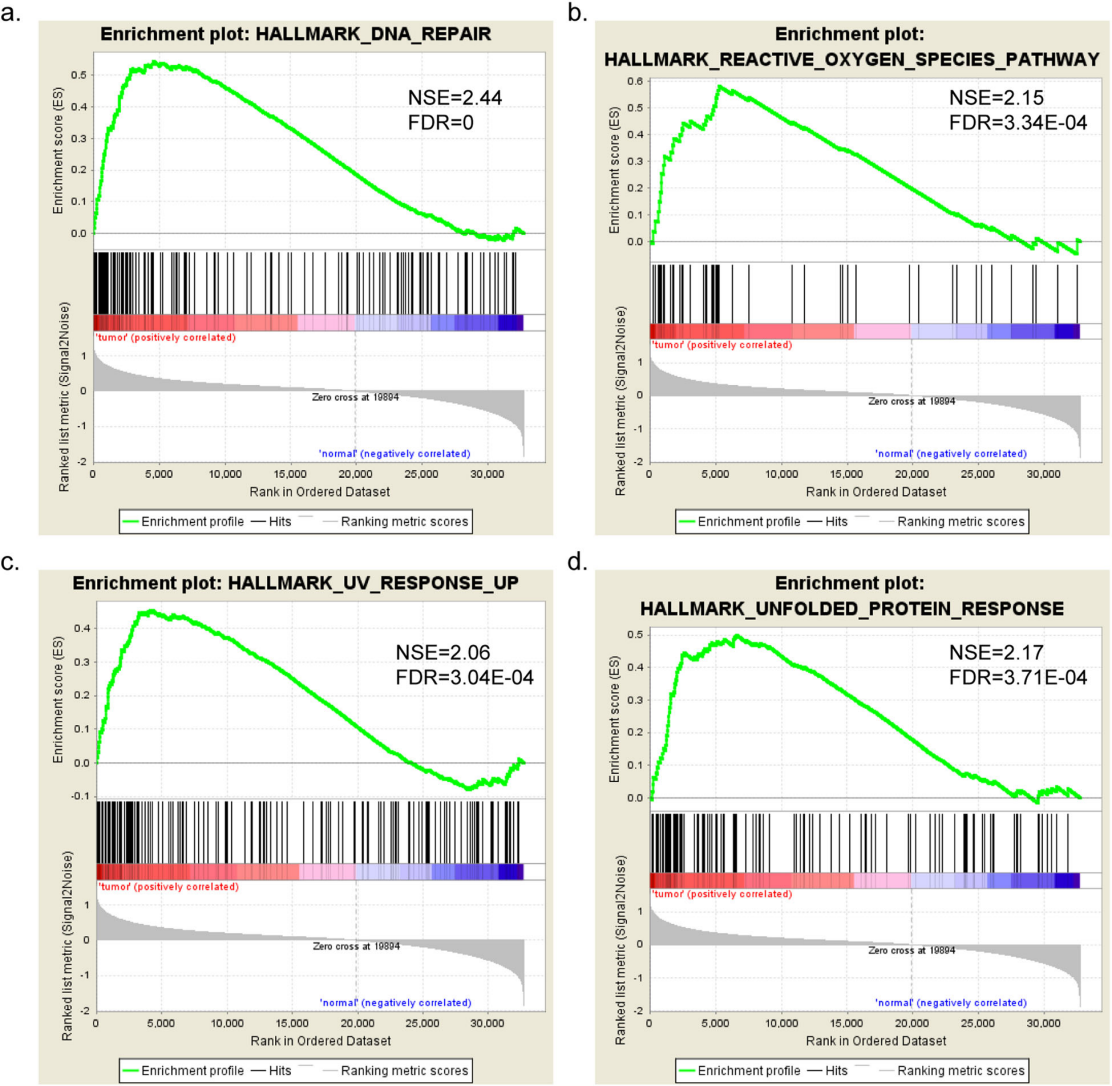
Four gene sets notably involved in the EC tumor phenotype using GSEA: (**a**) DNA repair, (**b**) reactive oxygen species pathway, (**c**) UV response up, and (**d**) unfolded protein response

The GSEA results showed that 4 of 50 gene sets (inclusion criteria: *p*-value < 0.05 and FDR < 0.05) were significantly enriched from the hallmark gene sets in the Molecular Signatures Database, including the DNA repair, unfolded protein response, reactive oxygen species pathway and UV response up (Fig. 1, Table 2**)**. Ranking by the *p*-value, the DNA repair process contained 142 key enriched genes, was selected for further research.

**Table 2:** Gene sets enriched in endometrial cancer TCGA samples

GS follow link to MSigDB	SIZE	NES	NOM ***p***-val	FDR q-val
HALLMARK_DNA_REPAIR	147	2.443	< 0.0001	< 0.0001
HALLMARK_UNFOLDED_PROTEIN_RESPONSE	111	2.169	< 0.0001	0.0004
HALLMARK_REACTIVE_OXYGEN_SPECIES_PATHWAY	47	2.149	< 0.0001	0.0003
HALLMARK_UV_RESPONSE_UP	157	2.056	< 0.0001	0.0003

### Identification of nine DNA repair-related genes and their characteristics

**Fig. 2:**
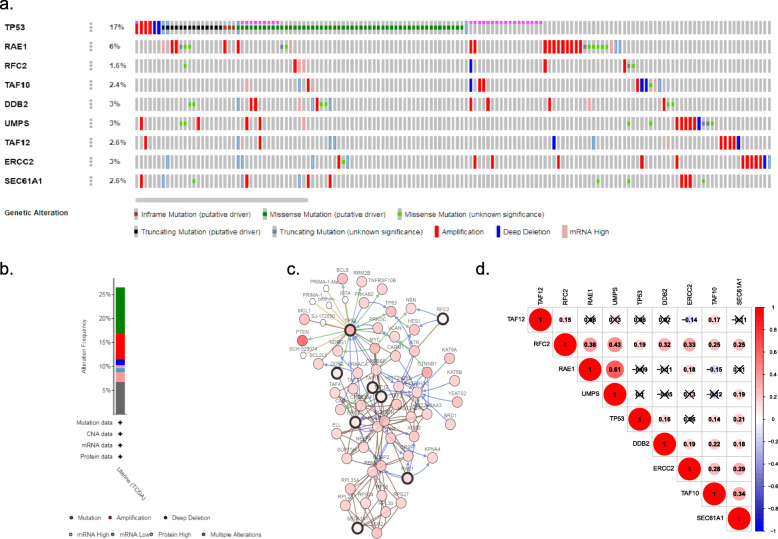
The gene mutation and correlation of 9 DNA repair-related genes analyzed through TCGA endometrial cancer patients. **a** Ten key genes are altered in 145 (27%) of queried patients. **b** The mutation types of 9 core genes. **c** The network instituted by 9 genes and the most sharply altered 50 neighbor genes. **d** The correlation efficiency between 9 genes

**Fig. 3:**
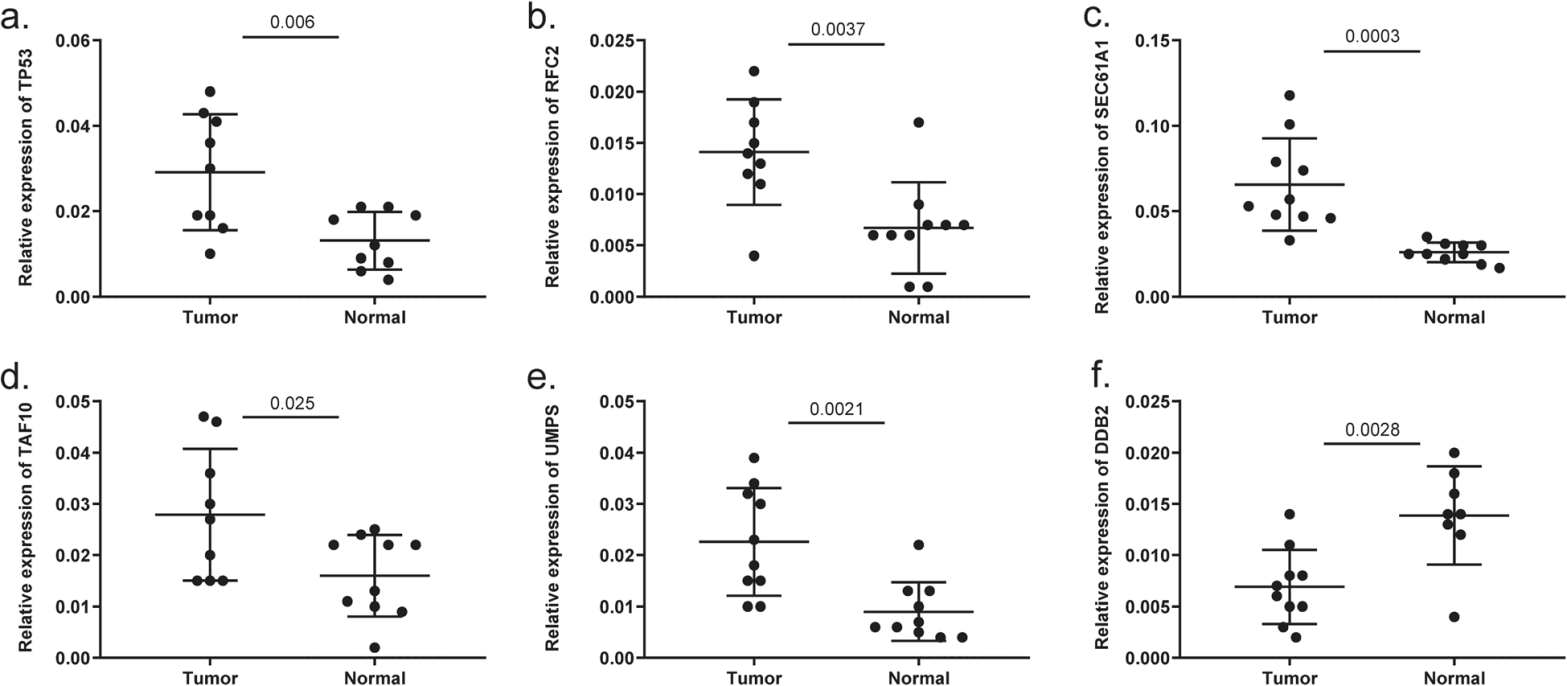
The expression level of (**a**) *TP53*, (**b**) *RFC2*, (**c**) *SEC61A1*, (**d**) *TAF10*, (**e**) *UMPS* and (**f**) *DDB2* mRNA in cancerous and normal tissues from the recruited EC patients

**Fig. 4:**
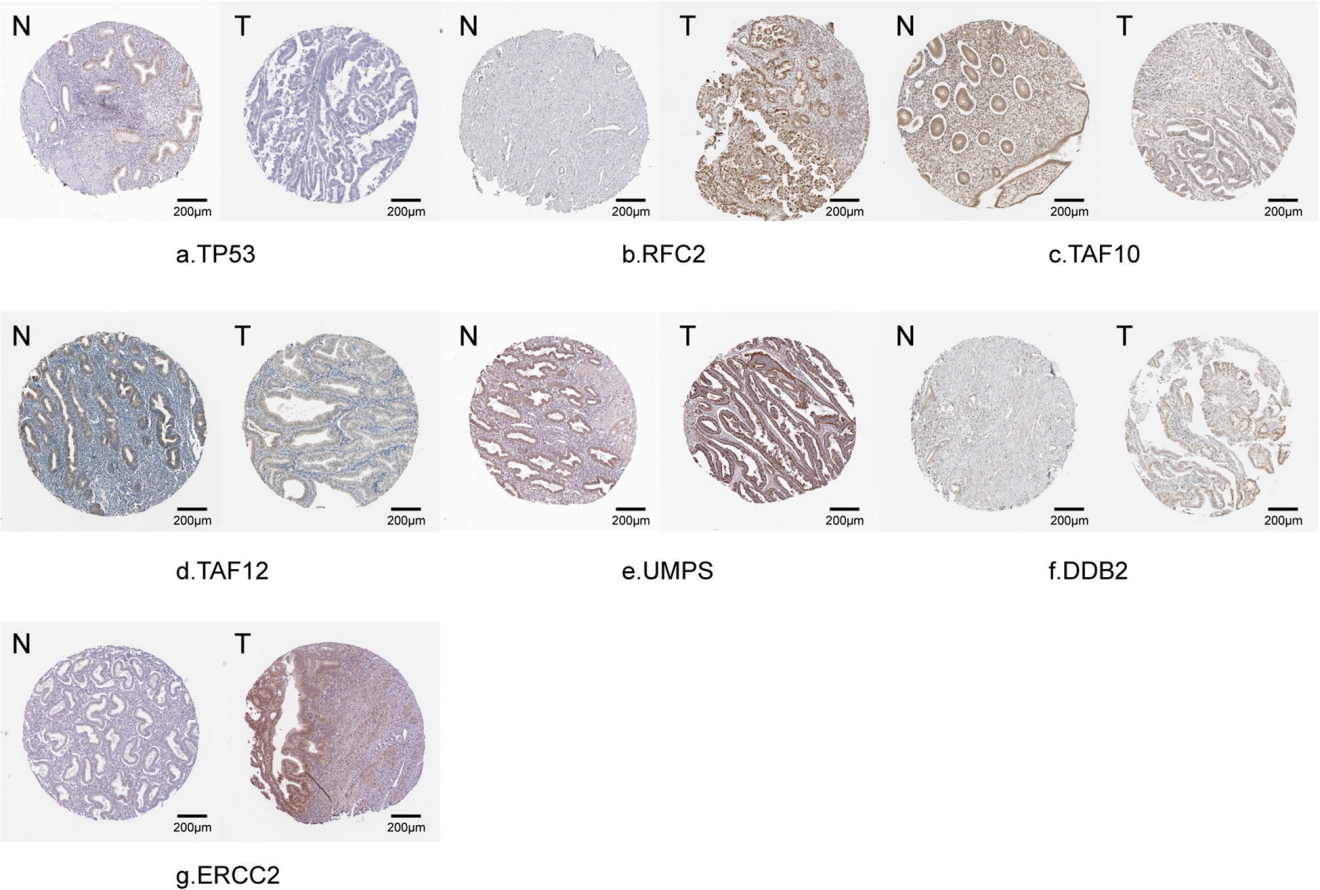
The expression level of (**a**) TP53, (**b**) RFC2, (**c**) TAF10, (**d**) TAF12, (**e**) UMPS, (**f**) DDB2 and (**g**) ERCC2 in cancerous and normal tissues from the HPA database

Through conducting univariate Cox regression analysis in the training cohort, a total of twenty-five genes were demonstrated to be associated with the OS of EC patients (*P* < 0.05)**.** Then, Lasso Cox regression analyses narrowed this figure into nineteen based on the minimum criteria (Fig. S1). Consequently, the multivariate Cox regression analysis identified 9 of 19 mRNA candidates to be significantly associated with survival, which were *tumor protein p53 (TP53)*, *ribonucleic acid export 1 (RAE1)*, *replication factor C 2 (RFC2)*, *TAF10 RNA polymerase II*, *TATA box binding protein (TBP)-associated factor (TAF10)*, *damage-specific DNA binding protein 2 (DDB2)*, *uridine monophosphate synthetase (UMPS)*, *TAF12 RNA polymerase II*, *TATA box binding protein (TBP)-associated factor (TAF12)*, *excision repair cross-complementation group 2 (ERCC2) and sec61 alpha 1 subunit (SEC61A1)*. By inputting all these genes into the cBioPortal website, we found that the gene alteration frequency of the 9 core genes among 548 EC samples was beyond 25% and these key genes were strikingly amplified and mutated. Intriguingly, the top 2 were *TP53* and *RAE1*, with *TP53* largely missense mutated and *RAE1* deeply amplified (Fig. 2a–c). The correlation efficiencies among the 9 candidate genes are shown in Fig. 2d. *RAE1* and *UMPS* had high correlation since the coefficient was 0.61. What’s more, Kaplan-Meier survival analysis manifested that *RAE1*, *UMPS* and *RFC2* were positively correlated to the OS of EC patients, while *TP53*, *DDB2*, *TAF10*, *SEC61A1* and *TAF12* were protective genes (Fig. S2). Based on the GEPIA web-based tool and the results from our recruited cohort, we found that *TP53*, *RFC2*, *TAF10*, *UMPS* and *SEC61A1* were overexpressed in EC patients, while *DDB2* was downregulated in cancer tissues (Fig. S3**,** Fig. 3). The expression level of proteins translated by most of these genes in EC tissues was also demonstrated by the HPA website (Fig. 4). Additionally, the relationships between the expression level of the 9 DNA repair-related genes and clinical parameters were also displayed in Table 3.

**Table 3:** Relationships between the expression level of 9 DNA repair-related genes and clinical factors in endometrial cancer

Gene	Age (<=60/> 60)		Stage (stage I & II/stage III & IV)		Histological Type (endometrioid/Mix & serous)		Grade (G1 & 2/G3 & 4)		Tumor Status (tumor free/with tumor)	
	T	*P*	T	*P*	T	*P*	T	*P*	T	*P*
TP53	1.346	0.179	2.301	**0.022**	3.083	**0.002**	−1.633	0.103	1.388	0.167
RAE1	−4.519	**7.70E-06**	−4.698	**4.67E-06**	−9.214	**1.51E-16**	12.663	**7.14E-32**	−3.39	**9.00E-04**
RFC2	−0.793	0.428	−2.505	**0.013**	−0.175	0.861	7.051	**5.77E-12**	−1.899	0.06
TAF10	2.012	**0.045**	1.114	0.266	2.112	**0.036**	−2.479	**0.014**	0.908	0.366
DDB2	2.995	**0.003**	2.206	**0.028**	7.618	**1.18E-12**	−3.72	**2.21E-04**	3.692	**3.13E-04**
UMPS	−2.821	**0.005**	−3.758	**2.21E-04**	−7.657	**1.03E-12**	10.782	**1.36E-24**	−3.526	**5.85E-04**
TAF12	2.886	**0.004**	1.035	0.302	0.034	0.973	1.224	0.221	0.637	0.525
ERCC2	−1.162	0.246	−0.906	0.366	−1.531	0.128	3.661	**2.79E-04**	−1.075	0.285
SEC61A1	0.783	0.434	1.427	0.155	4.914	**1.59E-06**	−1.144	0.253	1.491	0.138

Note: *T* T value of student’s t test; *P P*-value of student’s t test

### Development of a nine-gene model for survival prediction

**Fig. 5:**
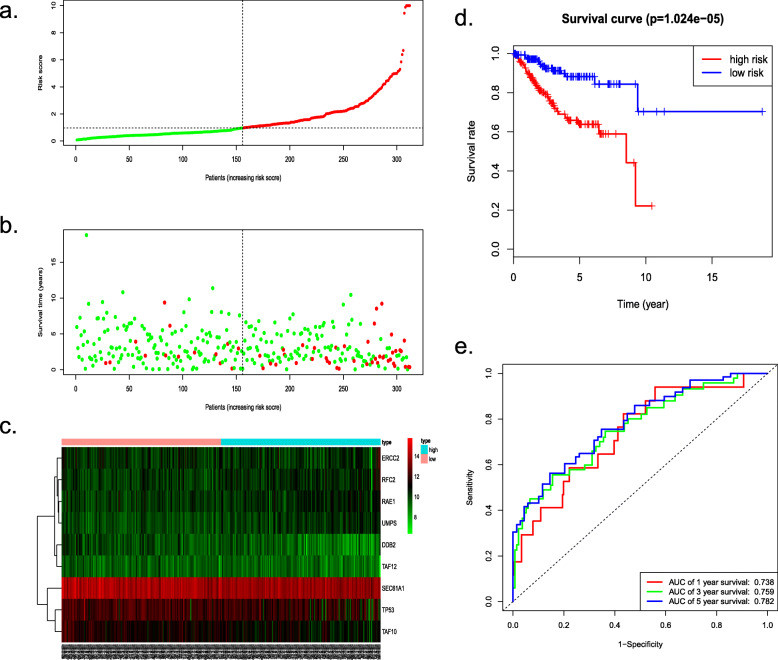
Survival analysis of the training cohort obtained from TCGA. **a** The risk score, (**b**) survival status, (**c**) expression heatmap, (**d**) Kaplan-Meier survival and (**e**) ROC curves to assess the prognostic value of the model in training TCGA cohort

To predict the prognosis of EC, we conducted a nine-gene-based risk score model as follows: risk score = (− 0.000113463 × expression level of *TP53*) + (− 0.000547732× expression level of *RAE1*) + (0.000969504× expression level of *RFC2*) + (− 0.000234564 × expression level of *TAF10*) + (− 0.001461235 × expression level of *DDB2*) + (0.000779442 × expression level of *UMPS*) + (− 0.001201708 × expression level of *TAF12*) + (0.000378192 × expression level of *ERCC2*) + (− 7.04E-05× expression level of *SEC61A1*). By this formula, we calculated the risk scores of individuals in the training cohort, as well as displaying the distribution of each patient’s survival status via dot plot (Fig. 5a, b). Additionally, patients from the training set were classified into a high- or low-risk group based on the median risk sccore. A heatmap was also constructed to display the expression pattern of each gene to visualize the variance of the high- and low-risk groups from the prognostic model (Fig. 5c). Specifically, Kaplan-Meier survival analysis demonstrated that the OS of the high-risk set was remarkably shorter than that of the low-risk set (*P* = 1.024e− 05) (Fig. 5d), ROC curves indicated the area under the ROC curve (AUC) of this prognostic model were 0.738, 0.759 and 0.782 at 1, 3, 5 years respectively (Fig. 5e). Furthermore, Univariate and multivariate Cox regression analyses verified this nine DNA repair-related gene-based model could be served as an independent indicator of EC (Fig. 7a, b).

### Validation of the prognostic model

**Fig. 6:**
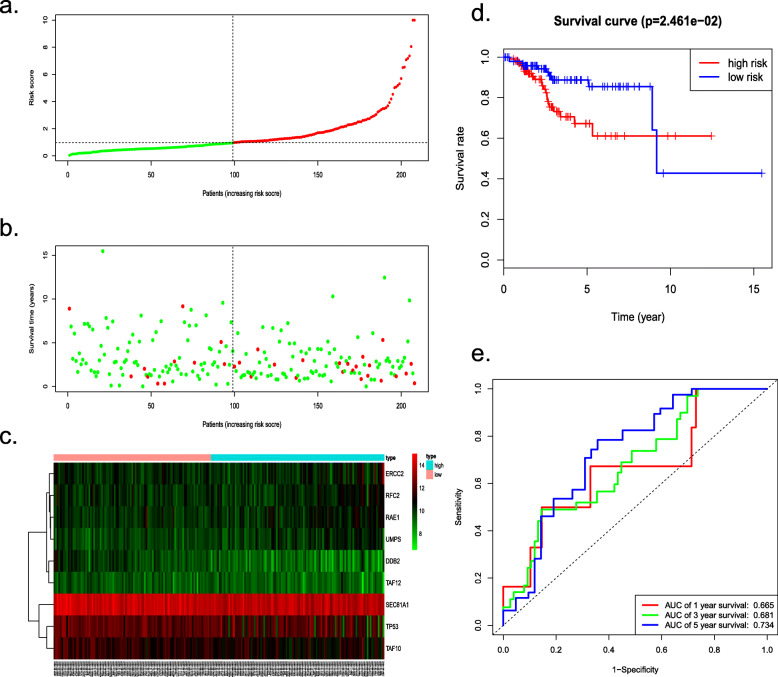
Survival analysis of the testing TCGA cohort. **a** The risk score, (**b**) survival status, (**c**) expression heatmap, (**d**) Kaplan-Meier survival and (**e**) ROC curves to assess the prognostic value of the model in the entire TCGA testing cohort

**Fig. 7:**
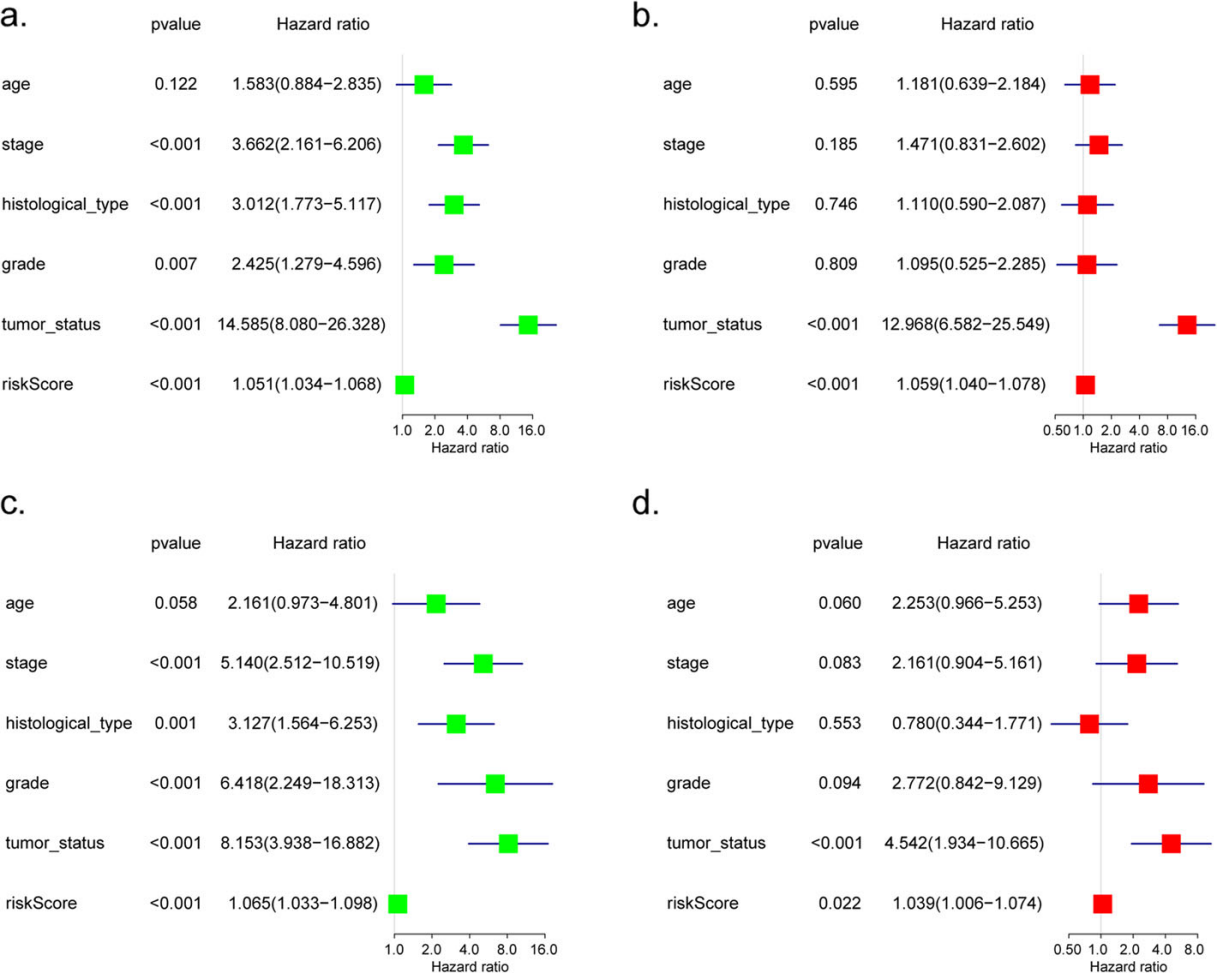
Univariate (**a**, **c**) and multivariate (**b**, **d**) cox regression analyses to test the independence of the model in the training (**a**, **b**) and testing (**c**, **d**) cohort, accompanied by clinicopathological characteristics

According to the cut-off value, we divided the testing cohort into 109 high-risk and 99 low-risk individuals. The risk scores and survival status of individuals were presented, as well as the expression levels of the 9 prognostic genes which was revealed by heatmap (Fig. 6a–c). The analysis of each patient from low- or high-risk group in the testing cohort were also executed. The Kaplan-Meier plot demonstrated the low-risk group exhibited better survival compared to the high-risk group (*P* = 2.461e− 02) (Fig. 6d). The 1-, 3-, and 5-year survival predictive accuracy of the model was 0.665, 0.681 and 0.734 respectively via ROC curve (Fig. 6e). Likewise, whether the prognostic model could be served as an independent indicator was also testified in the testing cohort through univariate and multivariate Cox regression, the analyses revealed the model could correctly predict the prognosis of EC patients without combining with other clinical characteristics (Fig. 7c, d).

**Fig. 8:**
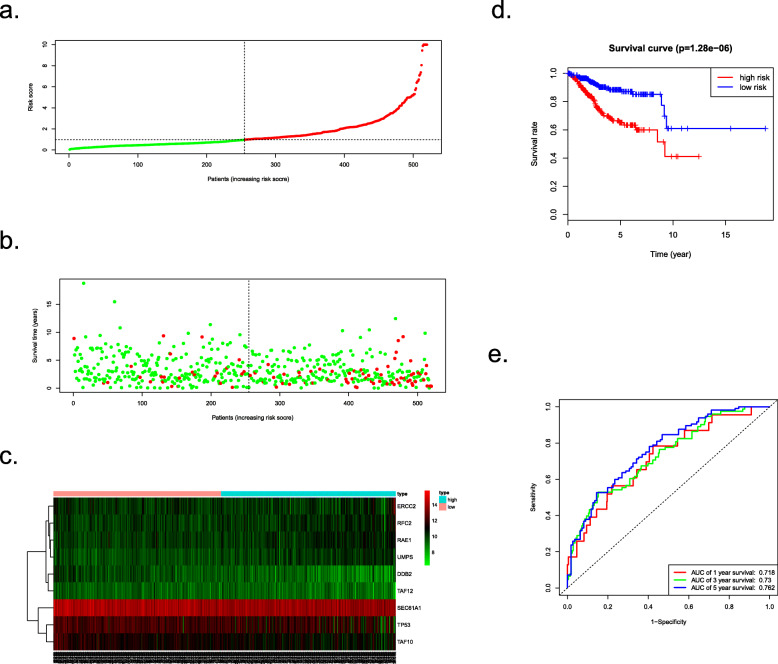
Survival analysis of the entire TCGA cohort. **a** The risk score, (**b**) survival status, (**c**) expression heatmap, (**d**) Kaplan-Meier survival and (**e**) ROC curves of the nine genes-based model for the entire TCGA EC cohort

**Fig. 9:**
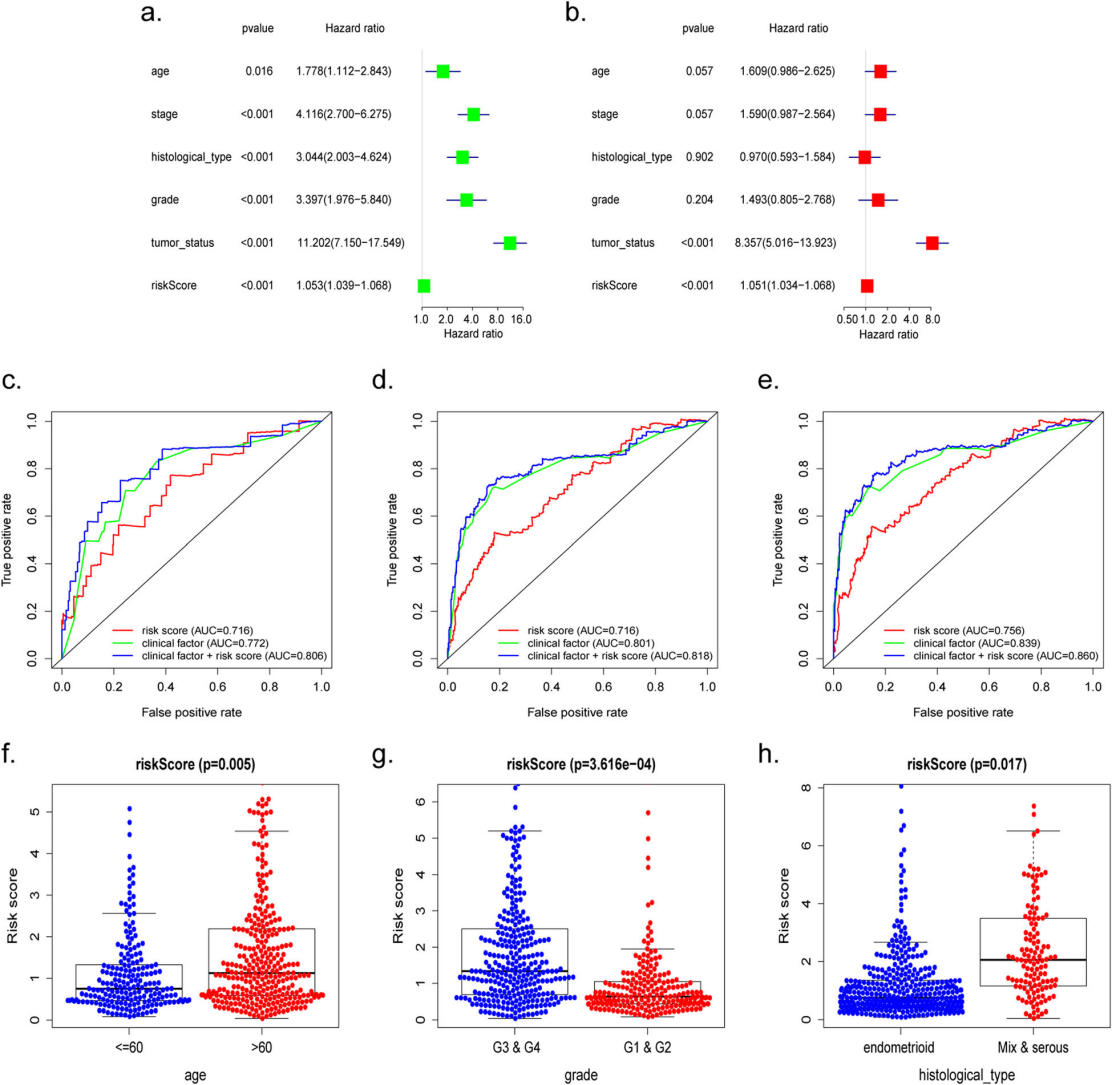
The prognostic value for the entire TCGA EC cohort was evaluated by univariate (**a**) and multivariate (**b)** regression and 1-, 3-, 5-year ROC (**c**–**e**) analysis. The relationship between the 9-mRNA signature and age (**f**), grade (**g**) and histological type (**h**) were also exhibited

The results of the survival analysis,based on nine-gene signature, about the prognosis of EC patients obtained from the entire TCGA cohort was parallel to the above results. Patients from the entire TCGA cohort were separated into a high-risk set (*n* = 270) and a low-risk set (*n* = 255) based on the median risk score. The distribution of each individual’s risk scores and survival status, as well as the expression levels of the 9 prognostic genes, were displayed (Fig. 8a–c). After that, Kaplan-Meier analysis showed that high-risk patients had shorter OS than low-risk patients (*P* = 1.28e− 06) (Fig. 8d). The ROC analysis was also made to reflect the predictive precision of the model in the TCGA cohort. The AUC of the prognostic model for 1-, 3-, and 5-year overall survival was 0.718, 0.73 and 0.762, respectively (Fig. 8e). Univariate and multivariate Cox regression analyses of the entire TCGA cohort demonstrated the independence of the model in predicting patient outcomes (Fig. 9a, b).

### Relationship between 9-gene-based model and clinicopathological features

The stratification analysis was performed using patient age, tumor grade, stage and histological type. The 9-mRNA model could still distinguish high-risk individuals either in stage I/II or stage III/IV subgroups, and the OS of the high-risk cohort was markedly inferior than that of the low-risk cohort in both stage I/II (*P* = 0.007976) and stage III/IV subgroups (*P* = 0.004892) (Fig. S4A, B). Likewise, the high-risk EC patients with the endometrioid type had a lower overall survival rate (*P* = 0.008215), which was acceptable to the results of the serous and mixed type subgroups (*P* = 0.040336) (Fig. S4C, D). The prognostic capability of the nine-mRNA model in patients with regard to grade and age were also validated. Patients ≥65 years old (Fig. S4F) or in the grade III/IV subgroup (Fig. S4E) were also divided into different cohorts. In parallel to the results above, the high-risk cohort in both subgroups was inclined to have worse OS. Intriguingly, the combination of the model and the clinical factors for predicting the 1-, 3- and 5-year OS of EC patients was full of high-efficient than anyone alone (Fig. 9c–e). Meanwhile, we compared the risk scores of patients according to age, grade and histological type. The results showed that patients with age > 60, G3/4 grade or mixed/serous histological type exhibited a higher risk (Fig. 9f–h).

### Diverse chemotherapeutic drug sensitivity of two subgroups

**Fig. 10:**
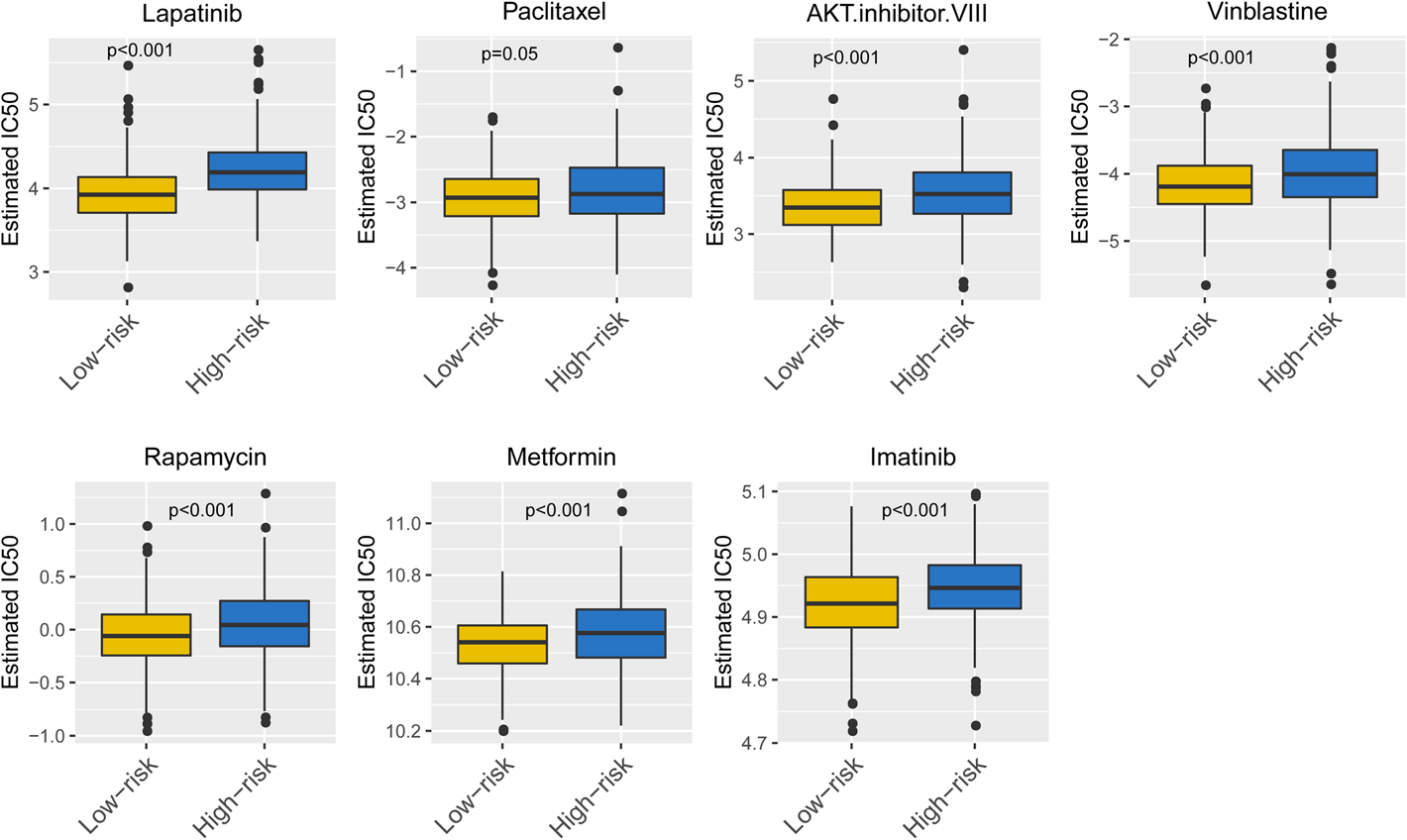
Differential putative chemotherapeutic and immunotherapeutic response of the high- and low-risk groups. The box plots of the estimated IC50 for cisplatin, paclitaxel, bleomycin, vinblastine, gemcitabine, rapamycin, metformin, imatinib, Akt inhibitor and lapatinib

As the chemotherapy is a common therapeutic strategy in EC treatment, we tried to assess the response of two subgroups to a list of common chemotherapeutic drugs. Thus, we trained the model on the GDSC cell line dataset by ridge regression with a satisfied predictive accuracy assessed by 10-fold cross-validation. We estimated the IC50 for each sample in the TCGA dataset based on the predictive model and observed significant differences in the estimated IC50 between the high- and low-risk subgroups for these chemotherapeutic drugs. Interestingly, the low-risk subgroup showed much higher sensitivity to paclitaxel, vinblastine, rapamycin, metformin, imatinib, Akt inhibitor and lapatinib than did the high-risk subgroup (Fig. 10).

### Nomogram construction and its clinical effectiveness

**Fig. 11:**
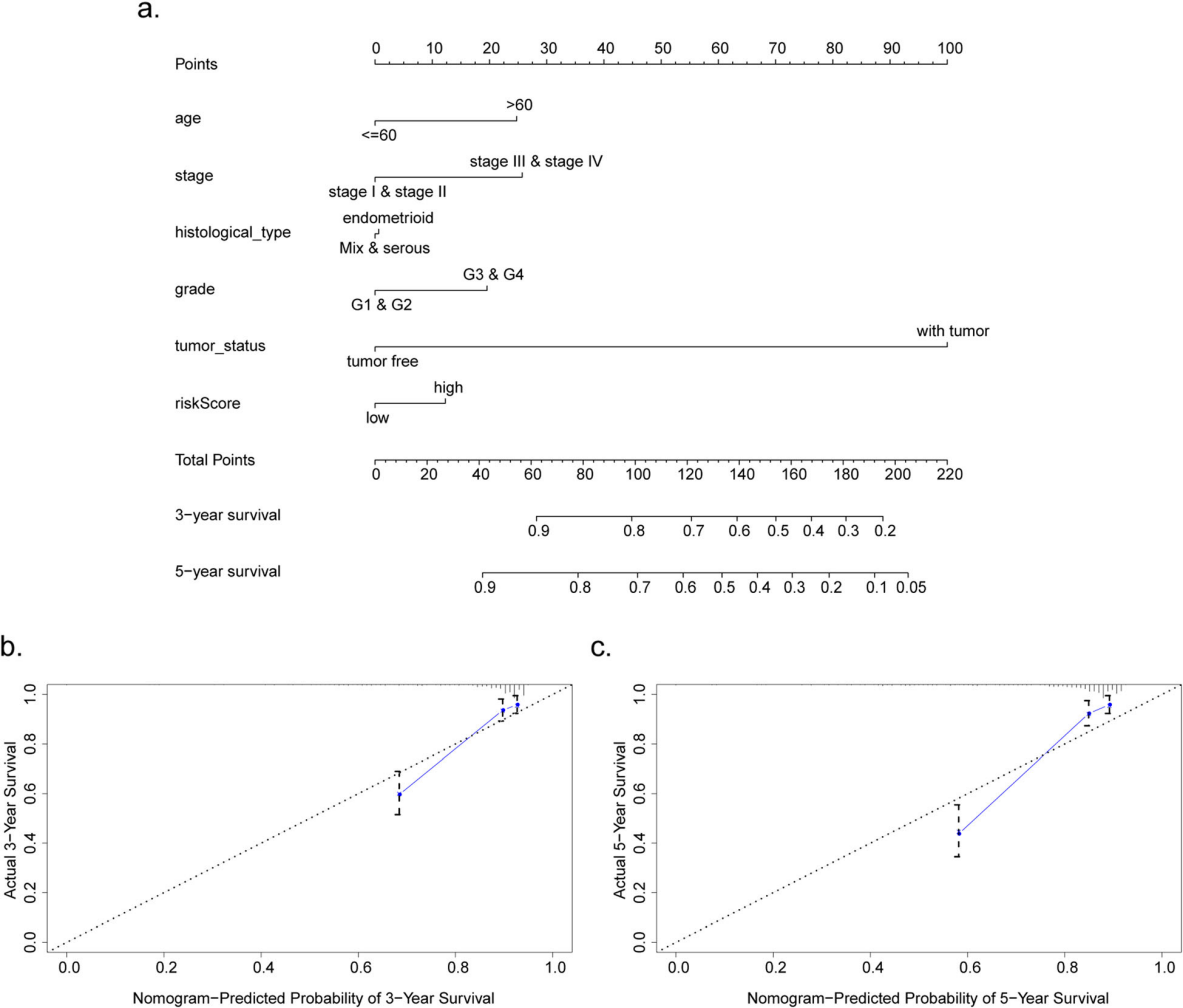
The nomogram to evaluate 3-, 5-year OS in the entire set. **a** The nomogram to evaluate the proportion of patients with 3- or 5-year OS. **b**, **c** The calibration plots to assess 3- or 5-year OS in patients. The nomogram is comprised of x-axis (represented the probability of the survival) and the y-axis (represented the actual survival)

To offer a measurable method for clinicians to estimate the OS of EC patients, we constructed a nomogram that contained the 9-mRNA-based model and other clinical characteristics, including age, tumor status, grade, stage and histological type. The 45° line represented the optimal prediction. The calibration curve of 3- or 5-year OS implied the nomogram could display a good performance with high concordance to the estimates (Fig. 11a–c).

## Discussion

In this study, we first performed GSEA on the mRNA profiles of all the EC patients from TCGA database and identified four significantly enriched biological processes: DNA repair, unfolded protein response, reactive oxygen species pathway and UV response up. We mainly focused on the DNA repair process, as it was the most eye-catching due to its *p*-value. DNA repair-associated genes have been characterized to be tightly associated with the physiological and pathological processes of various cancer types. Patients with metastatic castration-resistant prostate cancer resistant against prostate-specific membrane antigen (PSMA) targeting alpha-radiation therapy (TAT) often harbor mutations in *TP53*, *checkpoint kinase 2 (CHEK2)*, *breast cancer gene 1/2 (BRCA1/2)* and other DNA repair-associated genes [4]. The anti-tumor mechanisms of some chemotherapeutic drugs were partly due to their ability to interfere with DNA repair and induce DNA damage, whereas drug resistance often followed the disturbance of such processes. The DNA repair defects caused by loss of chromodomain helicase DNA binding protein 1 (CHD1) significantly enhanced prostate cancer therapeutic responsiveness [5]. Likewise, imipramine blue could suppress breast cancer growth and metastasis by inhibiting FoxM1-mediated DNA repair processes [13].

Next, we conducted univariate, lasso and multivariate Cox regression analyses, consequently, nine DNA repair-mRNAs were ascertained to be closely relevant to the OS of EC patients, which are *TP53*, *RAE1*, *RFC2*, *TAF10*, *DDB2*, *UMPS*, *TAF12*, *ERCC2* and *SEC61A1*. *TP53* mutation has been verified to act a crucial role in EC tumorigenesis. Kuhn et al. analyzed the genomes of 76 patients with uterine serous carcinomas and found frequent (81.6%) somatic mutations of *TP53* in the cohort [14]. Wild et al. also found that patients with p53 expression or mutation status showed poorer survival [15]. Wang et al. reported that the gain-of-function mutant p53-R248Q could effectively promote progression of EC by targeting the proteasome activator REGγ [16]. RAE1 is thought to participate in nucleocytoplasmic transport and attach cytoplasmic mRNPs to the cytoskeleton. RAE1 was found to be overexpressed in patients with estrogen receptor-positive breast cancer and related to poorer disease-free survival and distant metastasis-free survival. Upregulated RAE1 induced invasive and migratory phenotypes through mediating the epithelial-mesenchymal transition signals [17]. Herein, for patients with estrogen-dependent EC, RAE1 may serve as a novel candidate to be targeted for drug therapy. It has been widely studied that long-term tamoxifen usage could significantly induce an increased risk of EC and lead to worse prognosis [18, 19]. TAF10 was found to be closely associated with the formation of TFIID complexes and to be indispensable for folate receptor (FR)-alpha P4 promoter activity. Hao et al. discovered that E2 could downregulate various genes via direct TAF10-dependent association of the ER with the core promoter, while tamoxifen largely reverses this process [20]. The biological relationship between TAF10 and tamoxifen in the pathogenesis of EC still needs further study. Barakat et al. have found that overexpressed DDB2 in human ovarian cancer cells exhibited higher sensitivity to cisplatin through activating cellular apoptosis [21]. Zhao et al. uncovered a novel mechanism of DDB2 that could bind to the promoter region of NEDD4L to activate the TGF-β signaling pathway in ovarian cancer cells, finally improving the sensitivity of cells to TGF-β-induced growth inhibition [22]. Duffy et al. performed genome-wide screens in budding yeast and identified TDP1 and TAF12 as human orthologs recurrently overexpressed and/or amplified in human tumors, which cause chromosome instability (CIN) [23]. Likewise, Tong et al. performed a cross-species genome-wide search and recognized that TAF12, NFYC and RAD54L jointly participated in the development of choroid plexus carcinoma [24]. ERCC2, as a nucleotide excision repair (NER) gene, was found to be notably mutated in urothelial cancer and closely associated with the mutational activity of broad base changes [25]. In addition, the mutations of ERCC2 mediated the complete response to cisplatin-based chemosensitivity in muscle-invasive urothelial carcinoma [26]. These works provide new perspective that defects in NER can be exploited to enhance the efficacy of conventional platinum-based chemotherapy in the treatment of EC. RFC2 encodes a member of the activator 1 small subunits family, which could bind ATP and help promote cell survival. SEC61A1 is closely involved in membrane-bound ribosomes and the insertion of secretory and membrane polypeptides into the endoplasmic reticulum. Mutation or downregulation of UMPS could lead to 5-FU resistance during treatment and may serve as a marker predicting the toxicity of tegafur-uracil/leucovorin-based neoadjuvant chemoradiation for patients with locally advanced rectal cancer [27, 28]. However, such genes have yet not been studied in EC. In our study, we established a prognostic model involving these genes, uncovering the potential value of such genes in EC prognosis prediction.

Based on the calculated HRs and regression coefficients of each DNA repair-related gene, we constructed a prognostic model for survival prediction. Cox regression analysis manifested that the combination of nine DNA repair-related mRNAs has favorable efficacy and reproducibility in predicting prognosis of EC patients without relying on clinical parameters. Meanwhile, the relationship between the 9-mRNA signature and clinicopathological features was also investigated, which exhibited fine performance in distinguishing high-risk patients with age, tumor grade, stage and histological type.

EC is typically treated with surgery and platinum-based chemotherapy. By using the GDSC database, we found that patients in the low-risk subgroup exhibited more sensitive to commonly used chemotherapeutic agents compared with patients in the high-risk subgroup, which demonstrated that low-risk patients may benefit from the combination of chemotherapy. Finally, we constructed a nomogram built with a combined model to precisely predict the probability of OS for EC patients. The calibration curves showed the actual survival was highly consistent with the predicted survival, highlighting the excellent predictive value of the nomogram.

The strengths of our research lean on the following aspects. First, this study was highly methodologically reasonable, as it was mainly based on the TCGA public database that contained large amounts of samples. Second, all DNA repair-related prognostic genes were selected. The 9-mRNA model was confirmed as closely correlating to the prognosis of patients with EC and appropriate for prognostic estimation, since the results of survival analyses from both the training and testing cohorts were consistent with our hypothesis. The most innovative aspect of our study was the construction of a nomogram, which is the first model to combine the genetic data of patients with EC with clinical information to predict outcomes. Combined with traditional clinicopathological features, the predictive power of the nomogram is increased and may become routinely used in the future. However, some aspects of the current study should be improved. Further experimental studies are required to investigate the specific mechanisms regarding DNA repair-related genes in EC. In addition, the clinical value of the prognostic model and the nomogram need further validation in both clinical practice and prospective studies.

## Conclusion

The identified highly enriched gene sets may offer novel insight into the tumorigenesis and treatment of EC. In addition, the constructed 9-mRNA-based signature and nomogram had prominent clinical implications in prognostic estimation and tailored therapeutic strategy for EC patients.

## Supplementary Information

**Additional file 1: Fig. S1.** Identification of prognosis related mRNAs using LASSO regression model. (A) Plots of the cross-validation error rates. Each dot represents a lambda value along with error bars to give a confidence interval for the cross-validated error rate. (B) LASSO coefficient profiles of the mRNAs associated with the overall survival of endometrial cancer. **Fig. S2.** Kaplan-Meier plots of overall survival in two groups divided by each hub genes’ best-separation value. **Fig. S3.** The protein expression difference of (A) TP53, (B) RFC2, (C) SEC61A1, (D) TAF10, (E) UMPS and (F) DDB2 between cancerous and normal tissues from TCGA EC patients. **Fig. S4.** The 9-mRNA prognostic model distinguished high- and low-risk patients in different subgroups. (A) stage I/II subgroup, (B) stage III/IV subgroup, (C) endometrioid type subgroup, (D) serous and mixed type subgroup, (E) grade III/IV subgroup, (F) age ≥ 65 year-old subgroup.

## Data Availability

The datasets analyzed during the current study are available in the TCGA repository (https://portal.gdc.cancer.gov), the HPA repository (https://www.proteinatlas.org/), and the GDSC repository (https://www.cancerrxgene.org/).

## Abbreviations

EC: Endometrial cancer
FIGO: Federation internationale of gynecologie and obstetrigue
TCGA: The cancer genome atlas
GSEA: Gene set enrichment analysis
MsigDB: Molecular signatures database
FDR: False discovery rate
OS: Overall survival
HR: Hazard ratio
HPA: The human protein atlas
ROC: Receiver operating characteristic
GDSC: The genomics of drug sensitivity in cancer
IC50: Half-maximal inhibitory concentration
Ct: Cycle threshold
qRT-PCR: Real-time quantitative RT-PCR
TP53: Tumor protein p53
RAE1: Ribonucleic acid export 1
RFC2: Replication factor C 2
TAF10: TAF10 RNA polymerase II
TATA TBP: Box binding protein-associated factor
DDB2: Damage-specific DNA binding protein 2
UMPS: Uridine monophosphate synthetase
TAF12: TAF12 RNA polymerase II
TBP: TATA box binding protein-associated factor
ERCC2: Excision repair cross-complementation group 2
SEC61A1: Sec61 alpha 1 subunit
PSMA: Prostate-specific membrane antigen
TAT: Targeting alpha-radiation therapy
CHEK2: Checkpoint kinase 2
BRCA1/2: Breast cancer gene 1/2
CHD1: Chromodomain helicase DNA binding protein 1
FR: Folate receptor
NER: Nucleotide excision repair
CIN: Chromosome instability

## References

[1] Bray F, Ferlay J, Soerjomataram I, Siegel RL, Torre LA, Jemal A (2018). Global cancer statistics 2018: GLOBOCAN estimates of incidence and mortality worldwide for 36 cancers in 185 countries. CA Cancer J Clin.

[2] Yang JY, Werner HM, Li J, Westin SN, Lu Y, Halle MK, Trovik J, Salvesen HB, Mills GB, Liang H (2016). Integrative protein-based prognostic model for early-stage Endometrioid endometrial Cancer. Clin Cancer Res.

[3] Murali R, Soslow RA, Weigelt B (2014). Classification of endometrial carcinoma: more than two types. Lancet Oncol.

[4] Kratochwil C, Giesel FL, Heussel CP, Kazdal D, Endris V, Nientiedt C, Bruchertseifer F, Kippenberger M, Rathke H, Leichsenring J, et al. Patients resistant against PSMA-targeting alpha-radiation therapy often harbor mutations in DNA-repair associated genes. J Nucl Med. 2019;61(5):683-8. 10.2967/jnumed.119.234559 31601699

[5] Kari V, Mansour WY, Raul SK, Baumgart SJ, Mund A, Grade M, Sirma H, Simon R, Will H, Dobbelstein M (2016). Loss of CHD1 causes DNA repair defects and enhances prostate cancer therapeutic responsiveness. EMBO Rep.

[6] Mei J, Wang H, Wang R, Pan J, Liu C, Xu J (2020). Evaluation of X-ray repair cross-complementing family members as potential biomarkers for predicting progression and prognosis in hepatocellular carcinoma. Biomed Res Int.

[7] Mei J, Wang R, Xia D, Yang X, Zhou W, Wang H, Liu C (2020). BRCA1 is a novel prognostic Indicator and associates with immune cell infiltration in hepatocellular carcinoma. DNA Cell Biol.

[8] Subramanian A, Tamayo P, Mootha VK, Mukherjee S, Ebert BL, Gillette MA, Paulovich A, Pomeroy SL, Golub TR, Lander ES (2005). Gene set enrichment analysis: a knowledge-based approach for interpreting genome-wide expression profiles. Proc Natl Acad Sci U S A.

[9] Cerami E, Gao J, Dogrusoz U, Gross BE, Sumer SO, Aksoy BA, Jacobsen A, Byrne CJ, Heuer ML, Larsson E (2012). The cBio cancer genomics portal: an open platform for exploring multidimensional cancer genomics data. Cancer Discov.

[10] Uhlen M, Zhang C, Lee S, Sjostedt E, Fagerberg L, Bidkhori G, Benfeitas R, Arif M, Liu Z, Edfors F, et al. A pathology atlas of the human cancer transcriptome. Science. 2017;357(6352):eaan2507. 10.1126/science.aan2507 28818916

[11] Heagerty PJ, Zheng Y (2005). Survival model predictive accuracy and ROC curves. Biometrics.

[12] Geeleher P, Cox NJ, Huang RS (2014). Clinical drug response can be predicted using baseline gene expression levels and in vitro drug sensitivity in cell lines. Genome Biol.

[13] Rajamanickam S, Panneerdoss S, Gorthi A, Timilsina S, Onyeagucha B, Kovalskyy D, Ivanov D, Hanes MA, Vadlamudi RK, Chen Y (2016). Inhibition of FoxM1-mediated DNA repair by imipramine blue suppresses breast Cancer growth and metastasis. Clin Cancer Res.

[14] Kuhn E, Wu RC, Guan B, Wu G, Zhang J, Wang Y, Song L, Yuan X, Wei L, Roden RB (2012). Identification of molecular pathway aberrations in uterine serous carcinoma by genome-wide analyses. J Natl Cancer Inst.

[15] Wild PJ, Ikenberg K, Fuchs TJ, Rechsteiner M, Georgiev S, Fankhauser N, Noske A, Roessle M, Caduff R, Dellas A (2012). p53 suppresses type II endometrial carcinomas in mice and governs endometrial tumour aggressiveness in humans. EMBO Mol Med.

[16] Wang H, Bao W, Jiang F, Che Q, Chen Z, Wang F, Tong H, Dai C, He X, Liao Y (2015). Mutant p53 (p53-R248Q) functions as an oncogene in promoting endometrial cancer by up-regulating REGgamma. Cancer Lett.

[17] Oh JH, Hur H, Lee JY, Kim Y, Seo Y, Kim MH (2017). The mitotic checkpoint regulator RAE1 induces aggressive breast cancer cell phenotypes by mediating epithelial-mesenchymal transition. Sci Rep.

[18] Bergman L, Beelen ML, Gallee MP, Hollema H, Benraadt J, van Leeuwen FE (2000). Risk and prognosis of endometrial cancer after tamoxifen for breast cancer. Comprehensive Cancer Centres' ALERT Group. Assessment of Liver and Endometrial cancer Risk following Tamoxifen. Lancet.

[19] van Leeuwen FE, Benraadt J, Coebergh JW, Kiemeney LA, Gimbrere CH, Otter R, Schouten LJ, Damhuis RA, Bontenbal M, Diepenhorst FW (1994). Risk of endometrial cancer after tamoxifen treatment of breast cancer. Lancet.

[20] Hao H, d’Alincourt-Salazar M, Kelley KM, Shatnawi A, Mukherjee S, Shah YM, Ratnam M (2007). Estrogen-induced and TAFII30-mediated gene repression by direct recruitment of the estrogen receptor and co-repressors to the core promoter and its reversal by tamoxifen. Oncogene.

[21] Barakat BM, Wang QE, Han C, Milum K, Yin DT, Zhao Q, Wani G, el SA A, El-Mahdy MA, Wani AA (2010). Overexpression of DDB2 enhances the sensitivity of human ovarian cancer cells to cisplatin by augmenting cellular apoptosis. Int J Cancer.

[22] Zhao R, Cui T, Han C, Zhang X, He J, Srivastava AK, Yu J, Wani AA, Wang QE (2015). DDB2 modulates TGF-beta signal transduction in human ovarian cancer cells by downregulating NEDD4L. Nucleic Acids Res.

[23] Duffy S, Fam HK, Wang YK, Styles EB, Kim JH, Ang JS, Singh T, Larionov V, Shah SP, Andrews B (2016). Overexpression screens identify conserved dosage chromosome instability genes in yeast and human cancer. Proc Natl Acad Sci U S A.

[24] Tong Y, Merino D, Nimmervoll B, Gupta K, Wang YD, Finkelstein D, Dalton J, Ellison DW, Ma X, Zhang J (2015). Cross-species genomics identifies TAF12, NFYC, and RAD54L as choroid plexus carcinoma oncogenes. Cancer Cell.

[25] Kim J, Mouw KW, Polak P, Braunstein LZ, Kamburov A, Kwiatkowski DJ, Rosenberg JE, Van Allen EM, D'Andrea A, Getz G (2016). Somatic ERCC2 mutations are associated with a distinct genomic signature in urothelial tumors. Nat Genet.

[26] Van Allen EM, Mouw KW, Kim P, Iyer G, Wagle N, Al-Ahmadie H, Zhu C, Ostrovnaya I, Kryukov GV, O'Connor KW (2014). Somatic ERCC2 mutations correlate with cisplatin sensitivity in muscle-invasive urothelial carcinoma. Cancer Discov.

[27] Kim SY, Baek JY, Oh JH, Park SC, Sohn DK, Kim MJ, Chang HJ, Kong SY, Kim DY (2017). A phase II study of preoperative chemoradiation with tegafur-uracil plus leucovorin for locally advanced rectal cancer with pharmacogenetic analysis. Radiat Oncol.

[28] Griffith M, Mwenifumbo JC, Cheung PY, Paul JE, Pugh TJ, Tang MJ, Chittaranjan S, Morin RD, Asano JK, Ally AA (2013). Novel mRNA isoforms and mutations of uridine monophosphate synthetase and 5-fluorouracil resistance in colorectal cancer. Pharmacogenomics J.

