# Supplementary material for: Construction of a nine DNA repair-related gene prognostic classifier to predict prognosis in patients with endometrial carcinoma
Source: BMC Cancer. 2021 Jan 6;21:29. doi: 10.1186/s12885-020-07712-5 (PMC7789410; doi:10.1186/s12885-020-07712-5)
Supplement: Supplementary file 1 — Additional file 1: Fig. S1. Identification of prognosis related mRNAs using LASSO regression model. (A) Plots of the cross-validation error rates. Each dot represents a lambda value along with error bars to give a confidence interval for the cross-validated error rate. (B) LASSO coefficient profiles of the mRNAs associated with the overall survival of endometrial cancer. Fig. S2. Kaplan-Meier plots of overall survival in two groups divided by each hub genes’ best-separation value. Fig. S3. The protein expression difference of (A) TP53, (B) RFC2, (C) SEC61A1, (D) TAF10, (E) UMPS and (F) DDB2 between cancerous and normal tissues from TCGA EC patients. Fig. S4. The 9-mRNA prognostic model distinguished high- and low-risk patients in different subgroups. (A) stage I/II subgroup, (B) stage III/IV subgroup, (C) endometrioid type subgroup, (D) serous and mixed type subgroup, (E) grade III/IV subgroup, (F) age ≥ 65 year-old subgroup. [file 12885_2020_7712_MOESM1_ESM.docx]

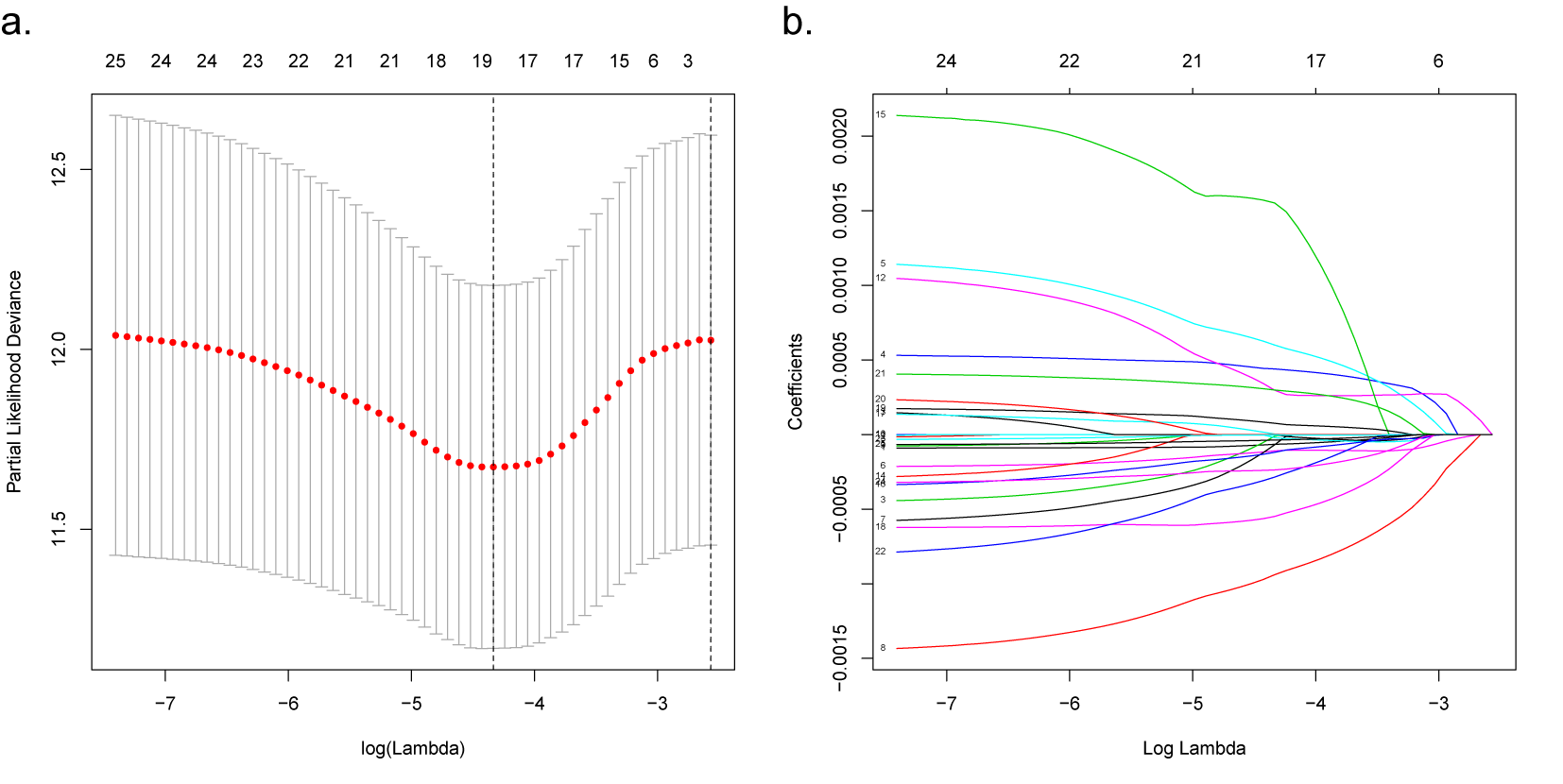


**Figure S1**. Identification of prognosis related mRNAs using LASSO regression model. (A) Plots of the cross-validation error rates. Each dot represents a lambda value along with error bars to give a confidence interval for the cross-validated error rate. (B) LASSO coefficient profiles of the mRNAs associated with the overall survival of endometrial cancer.


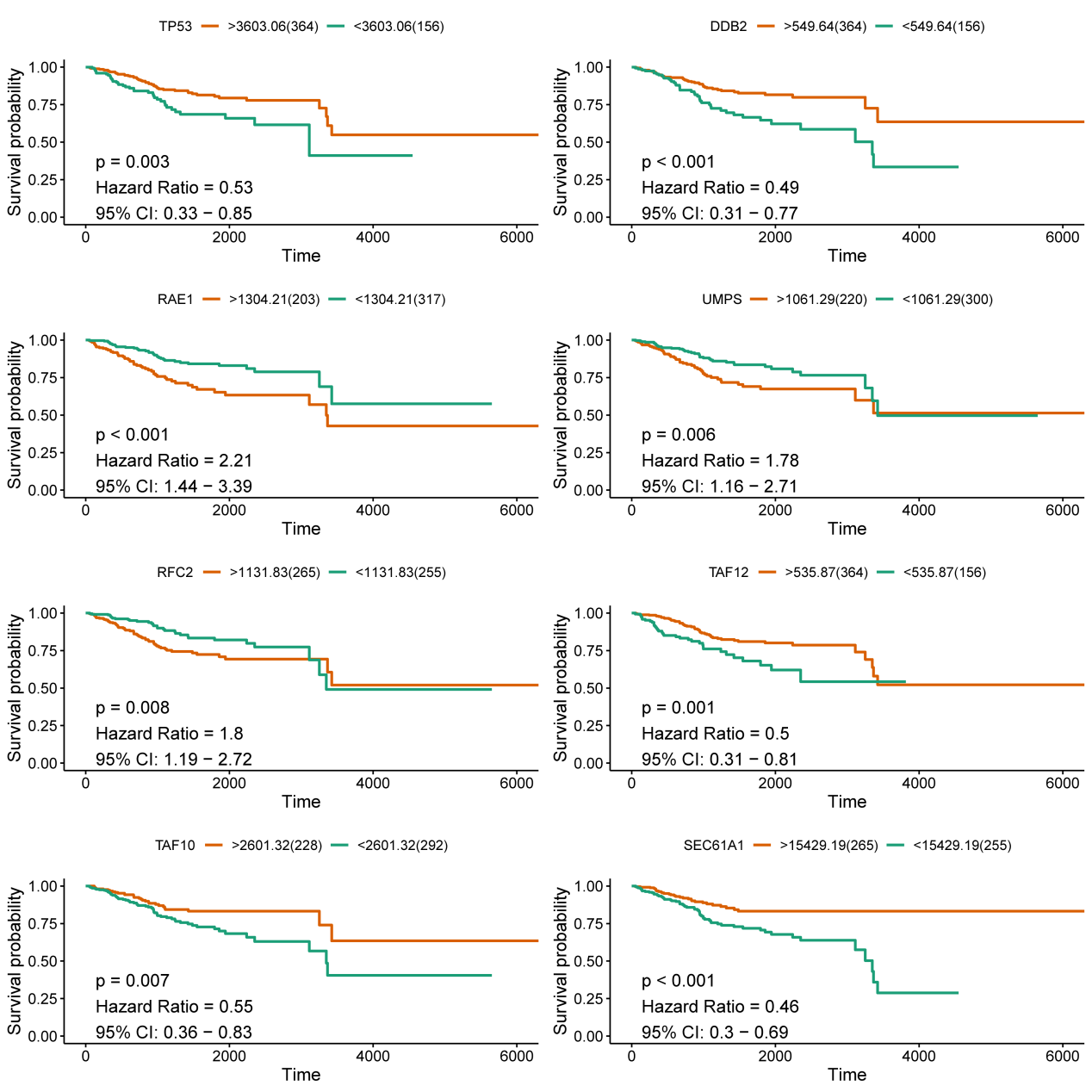


**Figure S2.** Kaplan-Meier plots of overall survival in two groups divided by each hub genes’ best-separation value.

**
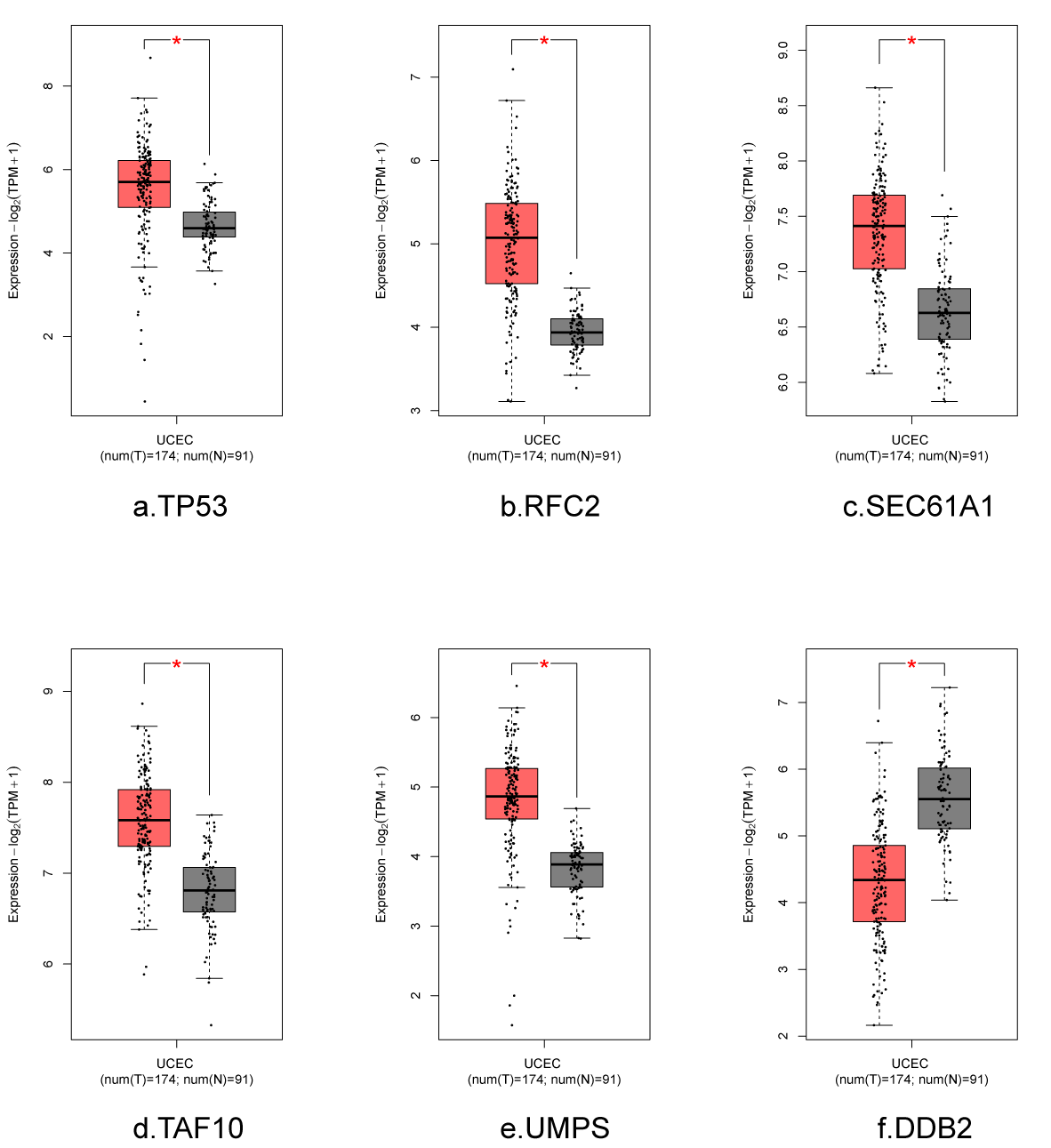
**

**Figure S3**. The protein expression difference of (A) TP53, (B) RFC2, (C) SEC61A1, (D) TAF10, (E) UMPS and (F) DDB2 between cancerous and normal tissues from TCGA EC patients.


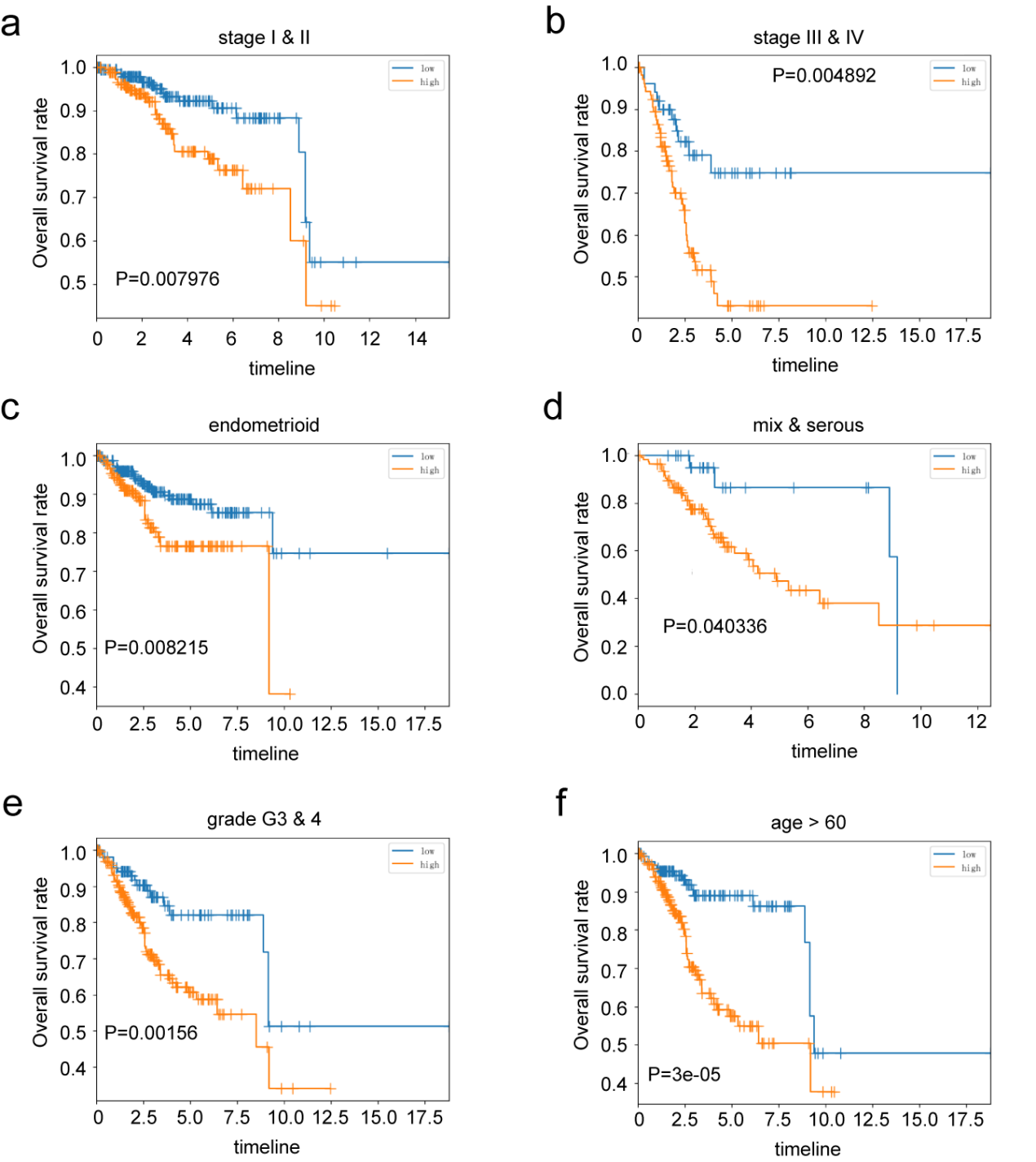


**Figure S4.** The 9-mRNA prognostic model distinguished high- and low-risk patients in different subgroups. (A) stage I/II subgroup, (B) stage III/IV subgroup, (C) endometrioid type subgroup, (D) serous and mixed type subgroup, (E) grade III/IV subgroup, (F) age ≥ 65 year-old subgroup.
